# An Audit: Compliance of Controlled Drug Prescriptions With Legal Requirements and Approved Home Office Wording

**DOI:** 10.1192/bjo.2024.624

**Published:** 2024-08-01

**Authors:** Ivan Saeger, Callum Sandhu, Michael Kelleher

**Affiliations:** South London and Maudsley NHS Foundation Trust, London, United Kingdom

## Abstract

**Aims:**

The completion of methadone and buprenorphine prescriptions, together: opioid substitution therapy (OST), must conform to legal requirements for the prescription of controlled drugs (CDs) as well as Home Office approved wording when writing instalment prescriptions.

Our service uses a part automated printing system for individual prescriptions and uses a manual record to track prescriptions issued for individual clients over time, a “script record”.

We aimed to audit the terms used on the internal script record as well to audit compliance of OST prescriptions with the legal requirements for CD prescriptions and Home Office Approved wording for instalments.

**Methods:**

All prescriptions for methadone or buprenorphine over the course of a week that were prepared for signing were audited.

The prescriptions were audited against the legal requirements for writing CD prescriptions and against Home Office approved wording for instalment prescriptions.

The script record was audited against internal standards for variation of terms used to describe frequency of collection of instalments.

**Results:**

A total of 64 prescriptions were audited.

100% of prescriptions complied with the legal requirements for the prescription of CDs.

7 prescriptions (11%) omitted Home Office approved wording to instruct what should be done on days when the dispensing pharmacist was closed, i.e. that instalments should be dispensed on the prior open day.

46 prescriptions (72%) had additional Home Office approved wording that was not applicable to the script. For example additional wording to allow for pickup of part of an instalment following a missed day, when the prescription was only for daily supervised consumption to begin with.

Audit of the internal script record found a total of 13 different terms used to describe frequency of collection of instalments; there are 6 standardised terms used within the internal script record. On 2 occasions the frequency of collection of instalments was left blank.

**Conclusion:**

It is essential that prescriptions for controlled drugs follow the legal requirements laid out for them; within the scope of our audit these were entirely adhered to.

There was however more variability in the use of the Home Office approved wording for instalments of OST. Scripts here tended to error for including additional wording not relevant to the specific script. Additionally, the service's own internal script record showed variability in the terms used to describe frequency of collection.

It is evidently important that the wording on prescriptions is clear and concise and the terms used internally are standardised.

